# Prevalence and factors associated with depression and suicidality among children and adolescents with sickle cell anaemia in eastern Uganda

**DOI:** 10.1007/s44192-026-00570-w

**Published:** 2026-08-09

**Authors:** Dedan Okello, Lisa Rynn, Milton W. Musaba, Sarah Kiguli, David Mukunya, Joseph Kirabira

**Affiliations:** 1 Faculty of Health Sciences, Department of Paediatrics and Child Health, Busitema University, P. O Box 1460, Mbale City, Uganda; 2 SEED Global Health Uganda, Kampala City, Uganda; 3 Faculty of Health Sciences, Department of Obstetrics and Gynaecology, Busitema University, P.O. Box 1460, Mbale City, Uganda; 4 Department Of Paediatrics and Child Health, Makerere University College of Health Sciences, P.O Box 7072, Kampala City, Uganda; 5 Faculty of Health Sciences, Department of Community and Public Health, Busitema University, P. O Box 1460, Mbale City, Uganda; 6 Faculty of Health Sciences, Department of Psychiatry, Busitema University, P. O Box 1460, Mbale City, Uganda

**Keywords:** Depression, Suicidality, Sickle cell anaemia, Children, Adolescents

## Abstract

**Introduction/background:**

Depression and suicidality are prevalent among individuals with chronic illnesses. Data on their prevalence in children and adolescents with sickle cell anaemia in Uganda are limited. This study aimed to determine the prevalence and associated factors of depression and suicidality among children with Sickle Cell Anaemia (SCA) in Eastern Uganda.

**Methods:**

This was a cross-sectional study among children with SCA between 9 and 17 years, at Mbale Regional Referral Hospital between 01/02/2024 and 05/06/2024. Depression and suicidality were measured using the MINI KID. Data were collected using an interviewer-administered questionnaire and entered into Kobo Toolbox for electronic data management. Analysis was performed using Stata 17. Bivariate and multivariable logistic regression analyses were done to examine factors associated with depression and suicidality. Statistical significance was assessed at α = 0.05.

**Results:**

421 participants were enrolled; the prevalence of depression was 31.8% (95% CI 27.4%–36.5%), while suicidality was 19.0% (95% CI 15.4–23.1%). The Factors associated with depression were parents' or guardians' employment. (aPR) 1.89, 95% CI 1.268–2.820, P value = 0.002). Household income over 150,000 Uganda Shillings monthly [aPR 0.56, 95% CI (0.327–0.97), P value = 0.039]. Parents' involvement in business [aPR 1.65, 95% CI (1.010–2.694), P value = 0.046]. 5–10 admissions [aPR 2.37, 95% CI (1.434–3.929), P value < 0.001]. For suicidality, being aged 13–17 years (aPR: 1.39 (95% CI 1.012–3.331) and a P = 0.012). Medication side effects (aPR: 1.49 (95% CI 1.017–3.024; p = 0.038).

**Conclusion:**

Children and adolescents living with SCA who had formally employed parents or guardians were significantly associated with depression. Older children and adolescents had a higher risk of suicidality. Findings highlight the magnitude of mental health challenges and underline the need for integrated mental health screening within chronic disease management.

## Introduction

Sickle cell anaemia is a permanent, debilitating condition of public health concern [1]. Globally, population-based prevalence of sickle cell anaemia is 0.008 among under-five children and 0.009 among those below 20 years [1]. In Sub-Saharan Africa, the burden of SCA remains noticeably high, where births in 2021 were 405 000 (95% UI 343 000–478 000), rising to 27·2% (23·4–30·2) since 2000 [2]. Nigeria, has the highest burden of SCA in sub-Saharan Africa, with over 150,000 children born with the disease [3]. Eswatini and Lesotho have the lowest burden of SCA in the region at 0.59% [4].Uganda's SCA burden remains relatively high at 13.3% of the population among children, with 15,000 to 20,000 babies born annually [5]. Of these children, (70–80)% if untreated die before 5 years of age [5]. In Eastern Uganda, the burden of SCA is high, with prevalence ranging between 1.5 and 2% [6].The chronicity of this condition comes along with a mental health burden [7].

Living with a chronic physical disease elevates the risk of developing psychiatric conditions, with studies indicating that individuals with chronic illnesses are 2 to 3 times more likely to experience depression or anxiety [8]. Chronic illnesses like cardiovascular diseases, cancer, metabolic, respiratory, chronic liver and degenerative disorders, plus chronic renal disease are responsible for 68.7% of stress, 51.1% of anxiety, and 58.8% of depression, respectively [9]. These chronic illnesses pose a significant risk to depression and suicidal tendencies [10]. Such similar challenges are more likely to occur among children living with SCA [11].

Depression is one of the most neglected mental health conditions among people living with sickle cell anaemia [12]. This usually predisposes people living with SCA to suicidal tendencies [13]. Globally, there is limited evidence showing the overall prevalence of depression and suicidality combined among children living with SCA. However, studies from different parts of the world, such as Jamaica, showed that 28.7% of adolescents were at risk of depression and 12.4% had attempted suicide [14]. In a Nigerian study among adolescents with SCA, 23.9% had depression, and 3.6% reported suicidality [15]. A Tanzanian study reported that 20% of children living with SCA were more likely to be depressed [15]. However, the study did not screen for suicidality in the study population [15]. In Congo Kinshasa, depression was observed in 86.4% among children with SCA aged 8 to 17, with 23.5% of them having had suicidal ideation [16].

In Uganda, depression was assessed in adult patients with SCA, where the prevalence of depression was up to two-thirds of the study population [17]. However, suicidality was not assessed [17]. In addition, the study was carried out among adult patients, leaving out children and adolescents [17].

While depression is prevalent among individuals with chronic illnesses, there is a paucity of data on depression and suicidality among children and adolescents with SCA in Uganda. Also, the few available studies that assessed depression among individuals with SCA used self-administered evaluation tools. Self-reported depressive symptoms scales have limitations in detecting major depression based on the Diagnostic and Statistical Manual of Mental Disorders (DSM-5-TR) criteria. In low-resource populations, these studies may not provide an accurate estimate of major depression prevalence in SCA [18]. This study was therefore aimed at assessing the prevalence and factors associated with depression and suicidality among children and adolescents with sickle cell anaemia in eastern Uganda.

## Methodology

### Study design

This study was a cross-sectional design to assess the prevalence and associated factors of depression and suicidality among children and adolescents with sickle cell anaemia (SCA) in Eastern Uganda. This design was chosen because it allows for a snapshot evaluation of the population at a single point in time, which is suitable for estimating prevalence.

### Study area

The study was conducted at Mbale Regional Referral Hospital in Eastern Uganda, which serves as a referral centre for several districts in the eastern region of Uganda. This study’s participants were from 17 districts in Eastern Uganda. These children and adolescents attend this clinic monthly for enrolment and follow-up services. Only children and adolescents attending the clinic were considered for the study. Patients at the clinic receive a range of services like clinical reviews and medications. For example, hydroxyurea, folic acid, and Fansidar (sulfadoxine 500 mg and pyrimethamine 25 mg). Some receive chloroquine for malaria prophylaxis and penicillin V for children under five years of age. All the services provided at the clinic are free of charge. We chose this site because it caters to more than 1,875 patients with sickle cell anaemia (SCA), mainly children and adolescents, but also adult patients. This clinic oversees the region’s satellite clinics in over 16 surrounding districts, making it ideal for our research.

### Study population

We targeted eligible children and adolescents with sickle cell anaemia, aged between 9 and 17 years, who had a confirmed HbSS DNA PCR diagnosis. Those who were attending the sickle cell clinic at Mbale Regional Referral Hospital. We also included those admitted to the ward with the same diagnosis but had improved clinically.

### Eligibility criteria

The study included children and adolescents aged 9 to 17 years who had been diagnosed with sickle cell anaemia using PCR at the Central Public Health Laboratory in Butabika, Kampala. Participants were recruited from the Sickle Cell Clinic at Mbale Regional Referral Hospital. This age range was chosen to encompass both childhood and adolescence, as defined by the MINI KID tool.

To be eligible for the study, participants needed to be clinically stable. Participants were expected not to be in a painful crisis or in need of emergency interventions, such as blood transfusions, at the time of enrolment. They were also required to be able to speak and understand the local language (Lumasaba, Luganda), into which the study materials were translated or the English language. And to provide assent with parental or guardian consent. Both forward and back translations of the tools were done by a professional agency (Varimetrics consultants’ translational services). In order to preserve psychiatric terms in the local languages, piloting of the tools was done before starting the data collection. Exclusion criteria included critical illness requiring intensive care, such as experiencing severe pain during data collection, or having cognitive impairment that would prevent participation.

### Sampling technique and sample size

The sample size was estimated by using the OpenEpi calculator [19]. We assumed a 50% proportion of depression and suicidality because there was no other existing study specific to our study population and a 95% confidence interval, resulting in 384 participants. An additional 38 participants (10%) were included to accommodate potential non-responses. Participants were consecutively sampled based on eligibility criteria until the entire sample size was achieved. The sampling process involved pre-screening all arrivals at the clinic for eligibility and the ability to consent and assent. Then, the research assistants enrolled eligible participants in order of arrival to prevent any perception of favouritism or bias. They assigned identification numbers to enrolled participants to prevent repeat enrolment by another research assistant during subsequent follow-up visits. This routine was maintained until the study enrolment target was met. Enrolment was done mainly during clinic days, and a few admitted participants were enrolled after improvement upon review on the wards. In this study, we approached 443 participants and only 422 accepted to be part of the study, creating a 95% response/ enrolment rate. This was sufficient for our desired sample size. One was later excluded during data analysis when it was discovered that they were outside the age range. This left a total of 421, which still allowed for 10% adjustment.

### Study variables

Our dependent variables were depression and suicidality, which were measured via the MINI KID [20]. The independent variables were age, sex, household income, education level, and frequency of admissions.

### Operational definition of study variables

*Sex:* sex was defined as the biological sex of the participant as recorded in the medical record or reported by the parent or guardian and categorised as male or female.

*Household income* was defined as the average monthly income of the participant’s household, as reported by the parent or guardian. It was categorised into < UGX10, 000. UGX 10,000–50,000, UGX 50,001–150,000, and > 150,000 per month.

*Education level:* Education level was defined as the highest level of formal education attained by the participant at the time of the study and categorised as no formal education, lower primary, upper primary, and secondary education.

*Frequency of admissions:* Frequency of admissions was defined as the number of hospital admissions related to sickle cell disease within the 12 months preceding the interview. It was categorised as none, fewer than five admissions, 5–10 admissions, 10–15 admissions, and more than 15 admissions.

### Recruitment and data collection procedure

Following the successful sampling of participants at the sickle cell clinic held every Wednesday of the week. Data collection started on 01/02/2024 and ended on 05/06/2024. The research assistants sought and completed a written consent and assent form from the participants and parents or guardians, respectively. They also obtained a signature or thumbprint from the participants during enrolment. The data collection tool was in three parts, namely: the demographic data section, the depression screening, and the suicidality section from the M.I.N.I. KID Tool. It was an interviewer-administered tool in hard copy, where the interviewer marked and recorded responses from enrolled participants.

Participants within the eligible age range were first identified, and the study was explained during clinic health education sessions. Consent from parents or guardians and assent from children were obtained. Non-participants continued receiving routine care. Consenting participants were interviewed in a private setting for 30–60 min, with confidentiality assured.

Trained research assistants (clinical officers and medical students) conducted interviews using the MINI-KID tool after receiving training from a psychiatrist. Data were then transferred to the electronic version of the Kobo tool for analysis.

### Data collection tools

We created a demographic section to capture participants' clinical data, borrowed from validated tools such as demographic health survey reports. For depression and suicidality, we utilised the M.I.N.I. International Neuropsychiatric Interview for Children and Adolescents (M.I.N.I KID), English version 7.0,2 [20], aligned with DSM-5 criteria. Originally designed for DSM-IV disorders, the MINI-KID has been updated for the DSM-5-TR and organised into diagnostic sections via a branching-tree logic that includes two screening questions for each disorder.

We were interested in sections A and B, which assessed depression and suicidality. To diagnose a current major depressive episode, a participant reported either depressed mood most of the day, nearly every day for the past two weeks, or markedly diminished interest or pleasure in most activities for the past 2 weeks. They should have had a total of 5 or more depressive symptoms (including one of the two core symptoms above) during the same 2-week period. And these symptoms should have caused clinically significant distress or impairment in daily activity.

For diagnosis of a Past major depressive Episode (Past MDE), a participant should not have met the above criteria for a current episode. They should have reported a previous period in their lifetime lasting at least 2 weeks with depressed mood and /or loss of interest and at least 5 depressive symptoms meeting MDE criteria. In other words, the depressive episode occurred previously but is not active now.

A participant had to be diagnosed with recurrent MDE if they had had at least one previous MDE before the current episode and there was a period of recovery/ remission between episodes (typically at least 2 months without significant depression or loss of interest).

Scores for suicidality are categorised as 1–8 points for low risk, 9–16 points for moderate risk, and above 17 points for high risk. The MINI-KID is a valuable tool with several noteworthy psychometric properties. It shows substantial to excellent concordance with the K-SADS-PL across various diagnoses, including mood disorders, anxiety disorders, substance use disorders, ADHD or behavioural disorders, and eating disorders. The tool demonstrated significant sensitivity for 15 out of 20 DSM-IV disorders and excellent specificity for 18 disorders. Overall, the MINI-KID provides reliable and valid psychiatric diagnoses for children and adolescents [21]. The same tool was used to validate the CRAFFT study, a tool in Kampala and Mbale, Uganda, that focuses on substance use disorders [22]. It has also been used in child mental health STC studies in Uganda and Burkina Faso [23]. The sociodemographic and clinical factors questionnaire was developed specifically for this study, guided by the national sociodemographic survey tools.

Both forward and back translations of the tools were done by a professional agency (Varimetrics consultants’ translational services). To preserve psychiatric terms in the local languages, piloting of the tools was done before starting the data collection.

### Data management and analysis

The data were downloaded from Kobo software into Microsoft Excel software and the dataset was examined for completeness before analysis. Records with missing data on variables included in the regression analyses were excluded using complete-case analysis because the proportion of missing data was minimal and was not expected to materially influence the study findings. It was then exported to Stata version 18 for analysis (http://www.stata.com/stata18/). Of the 422 enrolled participants, 421 were included in the analysis, representing 99.8% of those enrolled. One participant, 0.2%, was excluded because the participant did not meet the prespecified age eligibility criterion; this was not an exclusion due to missing data. Among the 421 participants included in the analysis, no additional observations were excluded from the regression models because of missing values; therefore, the proportion of missing data in the regression analyses was 0.0%. Continuous descriptive variables are presented as the means and standard deviations. Categorical variables are presented as proportions. We performed bivariable and multivariable modified Poisson regression to determine the associations between the explanatory variables and the outcome variables, depression and suicidality. Variables that demonstrated an association with the outcome at the bivariable analysis (p < 0.20), together with variables considered clinically or biologically relevant based on existing literature, were included in the multivariable models for depression and suicidality. (As long as they were not in the causal pathway and were not strongly collinear with other independent variables). These models were exploratory and hypothesis generating, without a predefined main exposure; consequently, confounding and interaction were not assessed. Categories with small cell counts were reviewed and, where clinically appropriate, combined to improve the stability and precision of the prevalence ratio estimates.

Collinearity was assessed using the variance inflation factor, and variables with a variance inflation factor greater than 10 were considered strongly collinear. Where collinearity was identified, the variable with greater conceptual and clinical relevance to the outcome was retained in the multivariable model.

A backward elimination approach was then applied cautiously to obtain a more parsimonious model from the set of variables identified a priori and from bivariable analysis, which included age, household income, employment status of the parents or guardians and medication side effects, while also assessing possible confounding. Variables removed from the model were re-evaluated for confounding and were retained if their exclusion changed the measure of association by 10% or more. Given the relatively limited number of outcome events, particularly for suicidality, care was taken to avoid overfitting by restricting multivariable models to variables considered most relevant based on prior evidence and observed associations.

### Ethical consideration

The study was approved by the Busitema University Research Ethics Committee (BUFHS-2023–140) and the Mbale Regional Referral Hospital Ethics Committee (MRRH-2023–140). Informed consent was obtained from parents or guardians, and assent was obtained from the participants, as all participants were under 18 (minors per Ugandan law). Trained research assistants collected data to prevent conflicts of interest. Confidentiality was maintained by securely storing participant information and assigning unique identification numbers. The electronic patient data were password-protected. A psychiatrist was involved in supervising the study and ensured that participants diagnosed with depression or suicidality received appropriate evaluation and care from the mental health professionals at Mbale Regional Referral Hospital, including counselling and pharmacologic treatment. The study adhered to the principles of the Declaration of Helsinki, prioritising the rights and well-being of participants.

All participants who screened positive for suicidality underwent an immediate assessment by the study psychiatrist to gauge the severity and urgency of the risk. Those identified as low or moderate risk were referred to the mental health clinic at Mbale Regional Referral Hospital for further evaluation and management. Participants at high or imminent risk received immediate intervention, including same-day evaluation, safety measures implementation, and urgent inpatient referrals when necessary. Given the involvement of minors, parents or legal guardians were informed of any identified risks confidentially, except when disclosure could increase risk. Caregivers were engaged in developing safety plans and accessing mental health services. For continuity of care, participants referred for mental health services were connected to the hospital’s psychiatry clinic for ongoing follow-up as per routine practice. All methods were carried out in accordance with relevant guidelines and regulations.

## Results

**Fig. 1:**
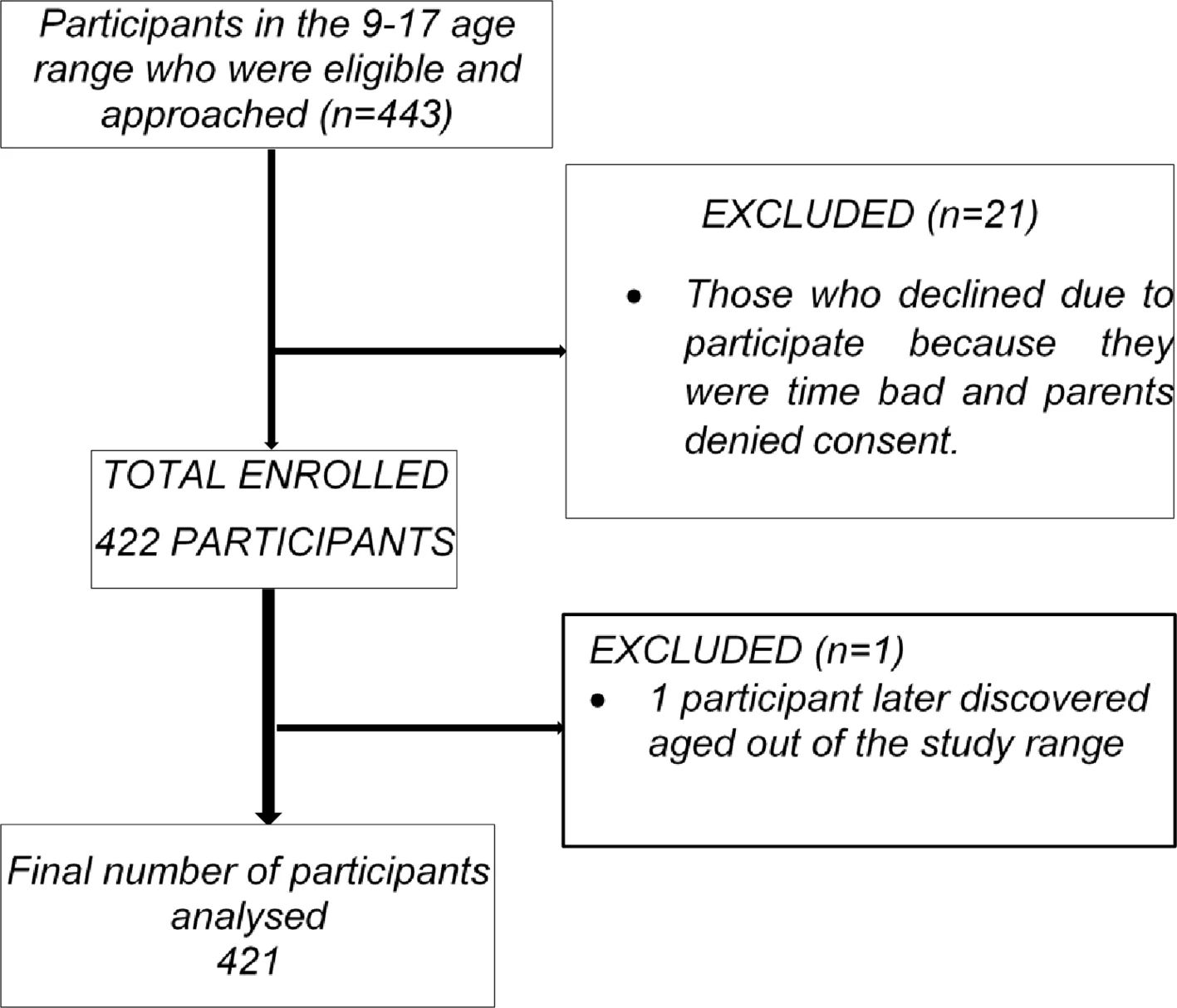
Participant enrolment in the study

A total of 443 participants were found eligible and approached to participate in the study out of the 760 participants aged between 9 and 17 years attending the clinic. 422 participants were enrolled in the study. 421 participants were successfully analysed after excluding one more participant on account of being 8 months below the required participant age category (Fig. 1).

### Sociodemographic characteristics of the participants

The children and adolescents in this study ranged from 9 to 17 years, with a mean age of 12.5 years and a standard deviation of 2.8 years. Female respondents (56.3%) were more than male participants (43.7%). Most participants (74.3%) lived with both parents; the majority of parents were unemployed (76.2%), and 43.7% of children were at the upper primary education level.

A higher proportion of children and adolescents (55.2%) aged 13–17 years were depressed. Children and adolescents aged 9–12 years had a lower prevalence of depression (44.8%). Those who had no education had the lowest rates of depression, with none reporting depressive symptoms. Children aged 13–17 years (62%) and those in upper primary education level had a high prevalence of suicidality. Details in Table 1

**Table 1:** Baseline sociodemographic and clinical characteristics of the participants stratified by depression and suicidality. (n = 421)

Variable	Total N = 42(%)	Depression		P-value	Suicidality		P-value
		No n = 287 (%)	Yes n = 134(%)		No n = 341 (%)	Yes n = 80 (%)	
*Gender*				0.345			0.257
Female	237 (56.3)	166 (57.8)	71 (53.0)		187 (54.8)	50 (62.5)	
Male	184 (43.7)	121 (42.2)	63 (47.0)		154 (45.2)	30 (37.5)	
*Age*				0.008			0.001
9–12	228 (54.2)	168 (58.5)	60 (44.8)		198 (58.1)	30 (37.5)	
13–17	193 (45.8)	119 (41.5)	74 (55.2)		143 (41.9)	50 (62.5)	
*Highest level of education*				0.001			0.001
No education	18 (4.3)	13 (4.5)	5 (3.7)		14 (4.1)	4 (5.0)	
Lower PRIMARY	183 (43.5)	141 (49.1)	42 (31.3)		163 (47.8)	20 (25.0)	
Upper primary	173 (41.1)	113 (39.4)	60 (44.8)		131 (38.4)	42 (52.5)	
Secondary	47 (11.2)	20 (7.0)	27 (20.1)		33 (9.7)	14 (17.5)	
*Staying with*				0.675			0.668
Both parents	313 (74.3)	214 (74.6)	99 (73.9)		255 (74.8)	58 (72.5)	
Guardian	37 (8.8)	23 (8.0)	14 (10.4)		31 (9.1)	6 (7.5)	
Single parents	71 (16.9)	50 (17.4)	21 (15.7)		55 (16.1)	16 (20.0)	
*Employment status of parent/Guardian*				0.119			0.128
No	320 (76.2)	225 (78.4)	95 (71.4)		265 (77.7)	55 (69.6)	
Yes	100 (23.8)	62 (21.6)	38 (28.6)		76 (22.3)	24 (30.4)	
*Major income-generating activity for your family*				0.849			0.446
Parents Formally employment	70 (16.7)	49 (17.1)	21 (15.8)		53 (15.6)	17 (21.2)	
Parents doing business	82 (19.6)	54 (18.9)	28 (21.1)		66 (19.5)	16 (20.0)	
Peasant	267 (63.7)	183 (64.0)	84 (63.2)		220 (64.9)	47 (58.8)	
*Estimated household monthly income*				0.12			0.661
< 10,000	31 (7.4)	16 (5.6)	15 (11.2)		26 (7.6)	5 (6.2)	
10,000–50,000	158 (37.5)	112 (39.0)	46 (34.3)		132 (38.7)	26 (32.5)	
50,001–150,000	101 (24.0)	69 (24.0)	32 (23.9)		81 (23.8)	20 (25.0)	
> 150,001	131 (31.1)	90 (31.4)	41 (30.6)		102 (29.9)	29 (36.2)	
*Number of admissions in the past 12 months*				0.010			0.496
None	52 (12.4)	37 (12.9)	15 (11.2)		45 (13.2)	7 (8.8)	
< 5	302 (71.7)	217 (75.6)	85 (63.4)		245 (71.8)	57 (71.2)	
5–10	58 (13.8)	28 (9.8)	30 (22.4)		44 (12.9)	14 (17.5)	
≥ 10	9 (2.2)	5 (1.7)	4 (2.7)		7 (2.1)	2 (2.4)	
*Experienced side effects*				0.159			0.033
No	399 (94.8)	275 (95.8)	124 (92.5)		327 (95.9)	72 (90.0)	
Yes	22 (5.2)	12 (4.2)	10 (7.5)		14 (4.1)	8 (10.0)	

Objective: To determine the prevalence of depression and suicidality among children and adolescents with SCD attending the clinic at Mbale Regional Referral Hospital

### Prevalence of major depression and suicide

One-third (31.8%, n = 134) (95% CI 27.4–36.5%) of the study children and adolescents reported experiencing major depression. The most frequently observed pattern was "past, recurrent" episodes, noted by 40.3% (n = 54) of the participants. Additionally, 21.6% (n = 29) had experienced depression in the past but were not currently depressed. Among the participants who had major depressive episodes, 35.8% (n = 48) were depressed at the time of the study.

With respect to suicidality, 19.0% (n = 80; 95% CI 15.4–23.1%) of the children and adolescents reported experiencing suicidal thoughts. 21.3% (n = 17) of the 80 participants stated that they had attempted suicide at least once, and 3.7% indicated that they might have suicidal thoughts in the near future. Furthermore, 21.2% (n = 17) of the sample points were categorised as very high severity for suicidality (> 17 points), whereas 55.0% (n = 44) scored lower points, 62.5% (n = 50) of the 80 participants who were suicidal had depression, with a P value < 0.001, as shown in Table 2.

**Table 2:** Prevalence of depression and suicidality among children with sickle cell anaemia at Mbale regional referral hospital (n = 421)

	Frequency	Percentage
*Major depression*		
No	287	68.2
Yes	134	31.8
*Major depressive episode*		
Past, current, and recurrent	43	32.1
Current	1	0.8
Past and current	4	3.0
Past	29	21.6
Past and recurrent	54	40.3
Recurrent	3	2.2
*Suicidality*		
No	341	81.0
Yes	80	19.0
*Type of suicidality*		
Current	60	75.0
Lifetime attempt	17	21.3
Likely in the near future	3	3.7
*Suicidality score*		
1–8points	44	55.0
9–16points	19	23.8
> 17points	17	21.2
*Suicidal behaviour disorder*		
No	413	98.1
Yes	8	1.9
*Suicidal behaviour disorder types*		
Current	4	50.0
In early remission	1	12.5
In remission	2	25.0
Lifetime attempt	1	12.5

Specific objective: To identify the factors associated with depression and suicidality in the study population

### Factors associated with depression among children with sickle cell anaemia

Children and adolescents whose parents or guardians were formally employed were 1.89 times as likely to experience depression as those whose parents or guardians were not formally employed. [Adjusted Prevalence ratio (aPR) 1.89, 95% CI 1.268–2.820, P value = 0.002]. Especially those whose parents were involved in business as formal employment with [aPR 1.65, 95% CI (1.010–2.694) and P value = 0.046].

Children and adolescents with 5 to 10 admissions were2.37 times as likely to experience depression as those who did not have any admissions in the past twelve months. [aPR 2.37, 95% CI (1.434–3.929), P value < 0.001].

Additionally, the children and adolescents from households with a monthly income of more than 150,000 Uganda Shillings (approximately 40.65 USD) were 0.56 times as less likely to experience depression as those from households with a monthly income of less than 10,000 Uganda Shillings (approximately 2.7 USD), with an [aPR 0.56, 95% CI (0.327–0.97), P value = 0.039], As shown in Table 3.

**Table 3:** Factors associated with major depressive episodes among children with sickle cell anaemia (n = 421)

Variable	Major depressive episodes		cPR (95% CI)	P value	aPR (95% CI)	P value
	No N = 287(%)	Yes N = 134(%)				
*Age*						
07–12	168 (58.5)	60 (44.8)	1		1	
13–17	119 (41.5)	74 (55.2)	1.46(1.099–1.931)	0.009	1.03(0.708–1.487)	0.890
*Education level*						
No formal education	13 (4.5)	5 (3.7)				
Lower primary	141 (49.1)	42 (31.3)	0.83(0.374**–**1.824)	0.637	0.7(0.323**–**1.526)	0.372
Upper primary	113 (39.4)	60 (44.8)	1.25(0.576**–**2.706)	0.574	1.05(0.487**–**2.265)	0.899
Secondary	20 (7.0)	27 (20.1)	2.07(0.943**–**4.536)	0.07	1.99(0.866**–**4.558)	0.105
*Parent/guardian formally employed*						
No	225 (78.4)	95 (71.4)	1		1	
*Yes	**62 (21.6)**	**38 (28.6)**	**1.99(1.126–2.732)**	**0.019**	**1.89(1.268–2.820)**	**0.002**
*Major income-generating activity for the family*						
Formally employed	49 (17.1)	21 (15.8)	1		1	
**Parents doing business**	**54 (18.9)**	**28 (21.1)**	**1.14(1.013–3.817)**	**0.008**	**1.65(1.010–2.694)**	**0.046**
Peasant	183 (64.0)	84 (63.2)	1.05(0.703**–**1.564)	0.816	1.65(0.969**–**2.802)	0.065
*Estimated household monthly income*						
< 10,000	16 (5.6)	15 (11.2)	1		1	
10,000 to 50,000	112 (39.0)	46 (34.3)	0.6(0.388—0.932)	0.023	0.7(0.439—1.112)	0.131
50,000 to 150,000	69 (24.0)	32 (23.9)	0.65(0.412**–**1.041)	0.073	0.68(0.409**–**1.117)	0.126
* > 150,000	**90 (31.4)**	**41 (30.6)**	**0.65(0.415–1.008)**	**0.054**	**0.56(0.327–0.97)**	**0.039**
*Number of admissions in the past 12 months*						
None	37 (12.9)	15 (11.2)	1		1	
< 5	217 (75.6)	85 (63.4)	0.98(0.614–1.552)	0.917	1.23(0.779–1.937)	0.375
*5 to 10	**28 (9.8)**	**30 (22.4)**	**1.79(1.093**–**2.94)**	**0.021**	**2.37(1.434**–**3.929)**	**0.001**
≥ 10	5 (1.7)	4 (3.0)	1.54(0.66–3.594)	0.317	2.23(0.97–5.129)	0.059

cPR-Crude Prevalence Ratios, aPR-Adjusted Prevalence Ratios, formally employed-Salaried earners, Parents doing business-Any payment for goods or services rendered by an individual, Peasant-a person who mainly depends on small-scale farming for livelihood

### Factors associated with suicidality among children with sickle cell anaemia

Children and adolescents aged 13 to 17 years were 1.39 times as likely to experience suicidality as those aged 9 to 12 years, with an aPR of 1.39 (95% CI 1.012–3.331) and a P value of 0.012. Additionally, children and adolescents who experienced side effects from medications were 1.49 times as likely to experience suicidality as those who did not experience any side effects (aPR: 1.49 (95% CI 1.017–3.024), with a P value of 0.038. In Table 4**.**

**Table 4:** Factors associated with suicidality among children with sickle cell anaemia (n = 421)

Variable	Suicidality		cPR (95% CI)	P value	aPR (95% CI)	P value
	No N = 341(%)	Yes N = 80(%)				
*Age*						
9–12	198 (58.1)	30 (37.5)	1		1	
^*^13–17	**143 (41.9)**	**50 (62.5)**	**1.97(1.306–2.968)**	**0.001**	**1.39(1.012–3.331)**	**0.012**
*Education level*						
No formal education	14 (4.1)	4 (5.0)				
Lower primary	163 (47.8)	20 (25.0)	0.49(0.188—1.284)	0.147	0.52(0.196—1.38)	0.19
Upper primary	131 (38.4)	42 (52.5)	1.09(0.442—2.699)	0.848	0.93(0.381—2.294)	0.883
Secondary	33 (9.7)	14 (17.5)	1.34(0.508—3.538)	0.554	1.02(0.382—2.743)	0.963
*Parent employed*						
No	265 (77.7)	55 (69.6)	1		1	
Yes	76 (22.3)	24 (30.4)	1.40(0.914–2.134)	0.123	1.22(0.614–2.43)	0.568
*Major income-generating activity*						
Formally employed	53 (15.6)	17 (21.2)	1		1	
Parents doing business	66 (19.5)	16 (20.0)	0.80(0.439—1.47)	0.478	0.89(0.412—1.909)	0.759
Peasant	220 (64.9)	47 (58.8)	0.72(0.445—1.182)	0.197	0.94(0.429—2.052)	0.874
*Experienced side effects*						
No	327 (95.9)	72 (90.0)	1		1	
^*^Yes	**14 (4.1)**	**8 (10.0)**	**2.02(1.115–3.642)**	**0.021**	**1.49(1.017–3.024)**	**0.038**

cPR- Crude Prevalence Ratios, aPR-Adjusted Prevalence Ratios, Formally employed-Salaried earners, Parents doing business-Any payment for goods or services rendered by an individual, Peasant-a person who mainly depends on small-scale farming for livelihood

## Discussion

This study assessed depressive symptoms, major depressive episodes, suicidality, and suicidal behaviour as interconnected but distinct constructs through the MINI-KID diagnostic interview. Depressive symptoms include emotional, cognitive, and behavioural indicators such as persistent sadness, loss of interest, sleep or appetite issues, fatigue, feelings of worthlessness, and concentration problems, regardless of meeting full diagnostic criteria. A major depressive episode is a clinically significant syndrome with a specific number and duration of symptoms within a certain timeframe, causing functional impairment and fulfilling MINI-KID criteria for depression. Suicidality encompasses suicidal thoughts and intentions, like ideation, thoughts of death, planning, and perceived risk, as assessed by the MINI-KID suicidality module. Conversely, suicidal behaviour involves actual self-injurious actions or suicide attempts, whether current or past, rather than just thoughts or plans. Thus, suicidality signifies suicidal thinking and risk, while suicidal behaviour indicates actual attempts, and both are distinguished from depressive symptoms and major depressive episodes.

Therefore, this study demonstrated a substantial mental health burden among children and adolescents with sickle cell anaemia (SCA) in Eastern Uganda, with notably high prevalences of depression (31.8%) and suicidality (19.0%). These findings underscore the profound psychological impact of living with a chronic, painful, and unpredictable condition [24]. The recurrent vaso-occlusive crises that can lead to stroke and severe pain causing functional impairment, frequent hospitalisations that lead to school absenteeism and low productivity, and the lifelong nature of SCA likely contribute to persistent emotional distress, reinforcing prior evidence that chronic illness in childhood is strongly associated with mood disorders [25].

The prevalence of depression observed in this study is higher than some reports from similar settings, such as studies in Nigeria,8.3% and 22%, respectively [26, 27], but lower than findings from the Democratic Republic of Congo at 86.4% [28]. These discrepancies may reflect methodological differences, including the use of more sensitive diagnostic tools such as the MINI-KID in our study, as well as contextual variations in healthcare access, socioeconomic conditions, and parental support. Environmental and economic constraints may further exacerbate depressive symptoms in this population.

The prevalence of suicidality (19%) is particularly concerning, especially given that a large proportion of affected participants (21.3%) had attempted suicide within the past year. This is slightly lower than the Congo Kinshasa study that showed 23.5% suicide ideation [28]. This may be related to unrecognised depressive symptoms, which may progress to a more severe outcome: suicidality [29].

Socioeconomic and family-related factors were also important determinants. Children and adolescents with formally employed parents or guardians, especially those in business, were associated with depression. This may be related to continued absence of their parents during the times of crisis and for moral and emotional support despite improved family income levels. Conversely, higher household income appeared protective, may be due to improved affordability of healthcare, pain management, and coping resources. This finding is similar to those from studies by Marsh et al., Adzika VA et al., and Ekoube et al*.,* [24, 30, 31]. This is also noted in both studies in America and Nigeria on the economic burden of sickle cell disease [32, 33].

The major clinical factor was frequent hospital admissions (5–10) that were associated with depression. This may be associated with the cumulative physical stress from Vasoclusive crises, severe pains and general ill health during the admissions. Psychological stress from school absenteeism, poor academic progress and non-productivity as expected by peers can exacerbate the depressive symptoms.

Older age in the children and adolescents, was significantly associated with suicidality. This may be related to prolonged suffering from crises, and related complications. Fear of death from an incurable disease and increased awareness of the complications can be part of the trigger. Also, the natural biopsychosocial stressors of adolescence, like hormonal changes and self-image formation issues could worsen the stress. This finding was noted in studies by Hassett et al. in 2014, Bhatt-Poulose et al. in 2016, and Carballo et al. in 2020 respectively [34–37].

Our findings also highlight the complex interplay between treatment-related factors and mental health. Medication side effects were significantly associated with suicidality. This may be due to the treatment burden, from taking routine hydroxyurea, Folic acid, and anti-malaria and pain medication. Also, frequent transfusions to correct severe anaemic states, may negatively influence adherence and psychological well-being in these patients, just as noted by Walsh K. E et al. in 2014 [38]. Although no specific medication causing side effects was identified in our study, the cumulative effect of multiple therapies may be associated to the distress and treatment fatigue like it was noted by Stübner et al. study among adolescents who were taking antidepressant medications [39]. Important to note, that the children and adolescents in this study, who attempted suicide, used routine medications provided at the clinic, just like those in the studies by Mishkin AD et al. and Swirsky et al. [13, 40].

Overall, these findings highlight the urgent need for integrated mental health services within SCA care programs. Routine screening for depression and suicidality, alongside psychosocial support for both patients and caregivers, is essential. Addressing socioeconomic disparities and treatment-related challenges may further mitigate the mental health burden in this vulnerable population.

### Strengths and limitations of the study

Our study was sufficiently powered by a large paediatric SCA sample, to examine the prevalence of depression and suicidality, as well as the various demographic, clinical, and economic factors associated with these issues. We also used a validated MINI-KID tool rather than only a symptom screening tool for the study process. This enabled us to include the screening for both depression and suicidality, widening our scope of study. The involvement of a competent psychiatrist gave us professional guidance in this study, and finally, the study high lights the relevance to SCA care in Uganda.

However, we encountered a few limitations in our research, namely, the cross-sectional design limits causal inference, the study population consisted only of patients who were actively seeking care (clinic-based sample), which excluded individuals who were not attending the clinic, this could affect the study results at the community level screening for those sickle cell anaemia patients who do not attend clinics. Children who were acutely ill were excluded which may underestimate depression or suicidality if acute illness is related to psychological distress. Additionally, there was the potential for recall bias among patients, although this was minimized by focusing on symptoms experienced over the past 12 months, which were readily available in their clinical records. Finally, we acknowledge that small cell counts may reduce the precision of the estimated prevalence ratios and contribute to wider confidence intervals as observed in the sparse categories, this was addressed through merging the small cell counts.

### Conclusion and recommendations

Older children and adolescents with sickle cell anaemia are at increased risk of suicidality, while those with formerly employed parents and guardians are at risk of depression. These were influenced by some factors such as; low household income status, frequent hospital admissions, and presence of medication side effects. These findings highlight the need for routine mental health screening and timely interventions in this population. Despite limitations of single-site sampling, our study included participants from multiple districts (17 out of 30), supporting the relevance of the findings. In a setting with limited mental health resources, the high burden observed underscores the urgent need for integrated psychosocial support for children with SCA in Eastern Uganda.

We recommend the update of Uganda's national policy for Sickle Cell Anaemia (SCA) management in children to include: Routine screening for depression and suicidality as soon as parents become unavailable and patients grow older. To offer psychological support, as well as emphasize close parental or guardian involvement in the follow up and reporting clinical outcomes of their children. Further research on the prevalence of depression and suicidality in other chronic diseases is highly recommended in this age category.

## Data Availability

The datasets generated during and/or analysed during the current study are available from the corresponding author on reasonable request.
